# Atypical spatiotemporal signatures of working memory brain processes in autism

**DOI:** 10.1038/tp.2015.107

**Published:** 2015-08-11

**Authors:** C M Urbain, E W Pang, M J Taylor

**Affiliations:** 1 Department of Diagnostic Imaging—Research, The Hospital for Sick Children, Toronto, ON, Canada; 2 Neuroscience & Mental Health Program, The Hospital for Sick Children Research Institute, Toronto, ON, Canada; 3 Neuropsychology and Functional Neuroimaging Research Unit at CRCN—Center for Research in Cognition and Neurosciences and UNI—ULB Neurosciences Institute, Université Libre de Bruxelles (ULB), Brussels, Belgium; 4 Division of Neurology, The Hospital for Sick Children, Toronto, ON, Canada; 5 Department of Psychology, University of Toronto, Toronto, ON, Canada

## Abstract

Working memory (WM) impairments may contribute to the profound behavioural manifestations in children with autism spectrum disorder (ASD). However, previous behavioural results are discrepant as are the few functional magnetic resonance imaging (fMRI) results collected in adults and adolescents with ASD. Here we investigate the precise temporal dynamics of WM-related brain activity using magnetoencephalography (MEG) in 20 children with ASD and matched controls during an *n*-back WM task across different load levels (1-back vs 2-back). Although behavioural results were similar between ASD and typically developing (TD) children, the between-group comparison performed on functional brain activity showed atypical WM-related brain processes in children with ASD compared with TD children. These atypical responses were observed in the ASD group from 200 to 600 ms post stimulus in both the low- (1-back) and high- (2-back) memory load conditions. During the 1-back condition, children with ASD showed reduced WM-related activations in the right hippocampus and the cingulate gyrus compared with TD children who showed more activation in the left dorso-lateral prefrontal cortex and the insulae. In the 2-back condition, children with ASD showed less activity in the left insula and midcingulate gyrus and more activity in the left precuneus than TD children. In addition, reduced activity in the anterior cingulate cortex was correlated with symptom severity in children with ASD. Thus, this MEG study identified the precise timing and sources of atypical WM-related activity in frontal, temporal and parietal regions in children with ASD. The potential impacts of such atypicalities on social deficits of autism are discussed.

## Introduction

Autism spectrum disorder (ASD) is a severe neurodevelopmental disorder characterized by striking impairments in social interaction and the presence of circumscribed interests and stereotyped-repetitive behaviours.^1^ Deficits in working memory (WM) and inhibition processes are key aspects of developmental psychopathology theories,^2^ as there is substantial evidence suggesting strong relations between WM difficulties and social or cognitive deficits in patients with ASD, and also schizophrenia or attention deficit hyperactivity disorder.^2, 3, 4, 5^ In that context, it has been suggested that WM difficulties might partly explain some symptoms of ASD, as a portion are related to poorer WM processes.^2, 6^ WM is the ability to store and manipulate information transiently, in the service of complex cognition and behaviour.^7^ It is not only strongly associated with academic achievement^8, 9^ but also has a central role in the online processing of complex cognitive information, such as social cognition and interpersonal interactions (for a recent review see Barendse e*t al.*^10^).

Behavioural studies have shown that ASD patients are particularly impaired in the spatial domain of WM,^6, 11, 12^ (but see Schuh and Eigsti^13^) and that their difficulties increase when the tasks impose heavier WM demands.^6, 14, 15, 16^ However, despite the importance of studying these processes in ASD, developmental research on WM functions in ASD is limited and overall findings are inconsistent as a number of studies have also found normal WM performance in this population.^17, 18, 19, 20^

At a neuroimaging level, a handful of functional magnetic resonance imaging (fMRI) studies addressing this topic have indicated atypical WM-related brain processes in ASD, usually in the absence of clear behavioural differences between clinical and control populations.^21, 22, 23^ In most studies, atypical frontal connectivity patterns or decreased activity in the prefrontal cortex have been reported in adults and adolescents with ASD in visuo-spatial WM tasks.^21, 22, 23, 24^ For instance, Silk *et al.*^23^ found WM processes involved in a mental rotation task were associated with reduced cortical activity of the anterior cingulate cortex (ACC), dorso-lateral prefrontal cortex (dlPFC) as well as the caudate nucleus in ASD compared with age-matched typically developing (TD) adolescents. Vogan *et al.*^25^ found markedly reduced frontal activity in children with ASD compared with controls in a WM task, also using fMRI.^25^ However, atypical brain responses associated with WM processes in ASD were not restricted to the frontal lobe. Using a cognitive control WM task, Solomon *et al.*^26^ showed that adolescents with ASD presented less frontal (BA 10) but also less parietal (BA 7 and BA 40) and occipital (BA 18) activation than TD participants. Likewise, reduced recruitment of the right posterior temporal regions^22^ in addition to reduced prefrontal activity^21, 22^ was identified in adults with ASD using two versions of the *n*-back task.

These atypicalities involved brain areas known to have a crucial role in WM processes. Whereas the prefrontal cortex acts as a control region, allowing the maintenance and manipulation of information in WM,^27, 28^ the inferior parietal lobe activity has been associated with an information buffer function^21^ and is associated with improved WM ability in healthy children.^29, 30, 31, 32^

Thus, these results suggest impairments in core WM processes yet compensatory strategies that allow, in the majority of cases, normative performance on specific WM memory tasks. However, compensatory brain processes do not rule out the possibility that individuals with ASD may be affected in more complex situations, according to the model that their WM deficits are related to specific disabilities in selecting appropriate processing strategies.^21^ Moreover, the poor temporal resolution of the fMRI studies reported above precludes an understanding of the timing of atypical WM components in ASD. Using magnetoencephalography (MEG), Hung *et al.*^33^ demonstrated significant WM-related brain processes involved in an *n*-back task occurring within the first few 100 ms after stimulus onset.^33^ Hence, MEG is a powerful technique that offers the ability to measure neuronal activity directly with millisecond time resolution, orders of magnitude higher than the time resolution of an fMRI (>1 s) and with excellent spatial resolution,^34, 35^ allowing the detection and the localization of weak transient activations.^35^ We apply this approach to improve our understanding of specific WM difficulties in children with ASD.

In addition, despite the central role of WM in social cognition and in cognitive development, very few fMRI studies have investigated WM-related brain processes in ASD during development.^23, 25, 26^ The present study addresses this gap and explores, at the behavioural and the neurophysiological level, the complex spatiotemporal processes underlying WM in children with ASD. To do so, we used MEG recordings during an *n*-back task to compare WM-related brain activity across different complexity levels (1-back vs 2-back) between children with high-functioning ASD and age-matched controls.

## Materials and methods

### Participants

This study included 20 children with high-functioning ASD and 20 TD controls that were age-, sex-, handedness and intelligence quotient matched. See Table 1 for demographic characteristics.

Participants were selected from a larger series of 38 children with high-functioning ASD and 26 TD control children (age range: 7y1mo—13y11mo). Children with autism were not included if they had an associated genetic or metabolic disorder, the presence of other neurological disorders, any current significant Axis I psychiatric comorbidities, medical illnesses, uncorrected vision and a learning disability or developmental delay as the primary diagnoses. Clinical diagnoses of ASD were confirmed in all cases with a combination of expert clinical judgment and the Autism Diagnostic Observation Schedule-General.^36^ TD children were not included if they reported a learning disability, developmental delay, any neurological, psychiatric or academic problem, as well as uncorrectable visual impairment. We arrived at our final sample of 40 children (20 per group) after sex- and age-matching and excluding children with excessive movement in the MRI and MEG scanners and inadequate task performance.

A further six children with ASD and one TD child had been tested, but their data were excluded as they performed the task at a chance level, meaning that their percentage of correct recognition (HITS) was equal or higher than the percentage of false alarm (FA), whatever the task condition. Of the six children with ASD, five were not able to perform the 2-back condition, whereas one ASD child failed to perform the 1-back condition. The excluded TD child was not able to perform both the 1- and the 2-back condition.

Children with ASD were recruited through community support centres, parent support groups and hospital advertisements; TD children were recruited through flyers and brochures posted at the hospital and the surrounding community. MEG and MRI scanning, as well as clinical and cognitive testing, were performed at the Hospital for Sick Children in Toronto. Experimental procedures were approved by the Hospital's Research Ethics Board. All children gave informed assent and the parents provided informed written consent.

### Experimental MEG task and procedure

**Figure 1:**
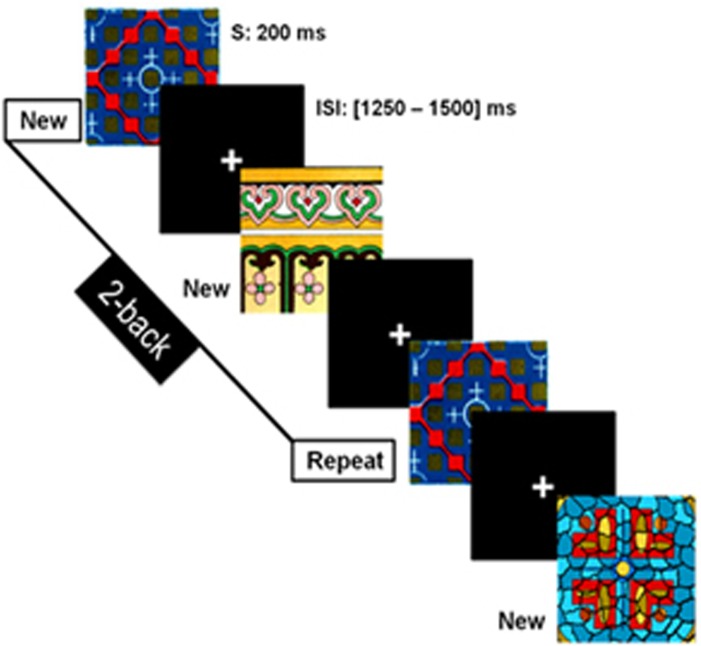
The *n*-back task. Schematic of the 2-back condition where the child is required to recognize that an image (Repeat) is the same as two images before (New; first occurrence of a picture). Each image (stimulus, S) appeared for 200 ms and was followed by a fixation cross displayed with an interstimulus interval (ISI) varying between 1250 and 1500 ms.

An *n*-back task requiring recognition of complex multi-coloured abstract images was used to investigate the WM ability of children with ASD. Children were instructed to press a key when they identified a repetition of a picture (target) presented 1 or 2 trials earlier (according to the 1-back or 2-back condition, see Figure 1).

Each *n*-back condition (1- and 2-back) was administered separately and counterbalanced across participants. All stimuli appeared on a projection screen located 80 cm from the children, where the visual angle of the stimuli subtended ~4° of their visual field. Each picture was shown for 200 ms followed by a fixation cross with an inter-stimulus interval varying between 1250 and 1500 ms. A photodiode was used to ensure accurate synchronization between the presentation of each visual stimulus and the trigger.

The 1-back condition had 230 trials, including 154 ‘NEW' trials (that is, first occurrence of a picture) and 76 ‘REPEAT' (target) trials, whereas the 2-back condition (which is more difficult due to the higher memory load) had 330 trials including 221 ‘NEW' and 109 ‘REPEAT' stimuli. A total of 375 different complex, coloured patterns were used across tasks, and there was no overlap of stimuli between the 1- and the 2-back conditions. Prior to entering the MEG, children were given a practice series to ensure that they understood the task and the two *n*-back conditions; this also gave them experience with the timing of the presentation for the stimuli. Stimuli used in the practice trials were not included in the experimental blocks.

### MEG data acquisition

MEG was recorded in a magnetically shielded room using a CTF MEG scanner with 151 axial gradiometers (Omega-151; MISL, Coquitlam, BC, Canada). Data were acquired at a sample rate of 600 s with a bandpass of 0–150 Hz. Head position inside the MEG dewar was measured before each recording session of each condition and continuously monitored using three tracking coils placed at the nasion and pre-auricular points. Coil placements were carefully measured and photographed in order to allow the off-line coregistration of the MEG data to the anatomical MRI of each child for source analyses.

### MRI data acquisition

Each child had a T1-weighted MRI (3D SAG MPRAGE: PAT, GRAPPA=2, TR/TE/FA=2300 ms/2.96 ms/90°, FOV=28.8 × 19.2cm, 256 × 256 matrix and 192 slices, slice thickness=1.0 mm isotropic voxels) from a 3 T MR scanner (MAGNETOM Tim Trio, Siemens, Erlangen, Germany), with a 12-channel head coil.

### Behavioural analyses

At the behavioural level, accuracy scores (percentage of correct recognition) (Acc), mean reaction times (RTs) and RT coefficient of variation (CV) (calculated for each subject as the s.d. of the mean RT divided by mean RT) associated with the target (repeat) stimuli were recorded in each *n*-back condition, and ‘repeat–correct' trials (RC) were compared between groups with repeated measures analysis of variance to ensure adequate quality of behavioural results prior to source analysis.

### Neuropsychological assessment

All children completed the Wechsler Abbreviated Scale of Intelligence as well as the Backwards Digit Recall, Listening Recall, Digit Recall, Mazes Memory and Block Recall subscales of the Working Memory Test Battery for Children (WMTB-C) to supplement behavioural data collected during the MEG task. Standardized scores on the subscales of the WMTB-C were compared across groups using repeated measures analysis of variance; age, sex and handedness were also compared across groups using *t*-tests for an independent sample.

### MEG analyses

With MEG we investigated the neurophysiological WM processes involved in correct recognition in the 1- and 2-back conditions. ‘Correct recognition effects', where RC trials elicited significantly stronger brain activity than New (N) trials have been described as a suitable comparison with identified WM-related brain activity.^33^ Such a contrast (RC>N) allows the identification of brain regions that are associated specifically with the recognition of the repeated (target) trial, excluding common brain activity shared between the N and RC conditions (for example, visual processing, baseline activity, etc).

Event-related fields associated with (1) correctly recognized target images (RC trials; please see Supplementary Figure S1) and with (2) ‘New' trials were recorded and analyzed within memory condition (1- vs 2-back) and then compared between groups (ASD vs TD). Memory load effects (1- vs 2-back) were then tested on between source effects (pseudo *z-*values) associated with correct recognition effects (RC–New) at the between-group level.

#### Preprocessing steps

MEG data were band-pass filtered at 1–70 Hz and time-locked to each image onset using a photodiode. Baseline-corrected epochs were then extracted from 200 ms prior to stimulus onset (baseline: −200 to 0 ms) to 1200 ms post stimulus onset. Epochs during which head movements exceeded 5 mm were rejected. Ocular and muscle artefacts were identified and subtracted from the trials on a subject-by-subject basis using ICA (ICA; EEGlab, http://www.sccn.ucsd.edu/eeglab^37^). ICA decomposition was performed simultaneously across all subjects and all conditions as recommended in the literature.^38^ For each participant, 30 components were examined for artefacts, and a minimum of two and a maximum of four components were removed per participant based on visual analysis of the component performed by an ICA expert.

#### Source reconstruction

Sources of MEG activity were localized using an event-related minimum variance vector beamformer, a method that allows precise localization of cortical sources and deep brain structures as previously demonstrated by our group using the same *n*-back task in adults.^33, 39^ Beamforming is a spatial filtering approach to MEG inverse source modelling that relies on a minimization of total brain power while constraining the gain in the voxel of interest, resulting in the suppression of background noise.^40^ Functional images of whole-head activity were produced in each condition (RC vs New) by applying beamformer weights on averaged 50-ms time intervals over the task epoch of interest (0–600 ms). Weights were derived using both a forward field (a model of the fields measured in response to a unit current with known location/orientation) and an estimated channel-level covariance matrix. Head modelling was computed using a multisphere headmodel^41^ fitted to the inner skull surface derived from each child's MRI using FSL's brain extraction tool (http://www.fmrib.ox.ac.uk/fsl/bet2/index. html). Volumetric images of brain activations were then normalized into Montreal Neurological Institute space using ANTS (http://picsl.upenn.edu/software/ants/) with a 5-mm voxel-grid of source power^42, 43^ viewable in AFNI software (http://afni.nimh.nih.gov/afni/). Group maps were generated by averaging functional images on a time-point-by-time-point basis for all individuals.

### Statistical analyses

Resulting functional beamformer images associated with RC and New trials were statistically compared for each memory load (1- and 2-back) separately with a non-parametric, paired random permutation test^43^ (3000 permutations) to compare the temporal and spatial dynamics of correct recognition (Repeat–Correct) effects (RC>New) obtained within the group. For voxels of significant activations, we computed source time courses (also called virtual sensors) and estimated differences between each time point across individuals using a non-parametric, paired random permutation test (3000 permutations) to identify significant differences in brain activity over time between conditions (RC vs New) in each group. Statistical significance of within-group comparisons for the significant voxels of activations was set at *P*<0.05, corrected for multiple comparisons in the whole source–space volume using a Sidak Correction.^44^

Source effects associated with correct recognition effects (*P*^corr^<0.05) at the within-group level were then submitted to a between-group comparison analysis both at the time and space levels. To do so, the three-dimensional images for the New stimuli were subtracted from the images of the RC correct stimuli to compare correct recognition effects between TD and ASD children using permutation tests. Between-group differences on the subtracted beamformer images were tested as well using a non-parametric (3000 permutations), unpaired random permutation test,^43^ and statistical significance for the significant voxels of activations was set at *P*<0.005.

*T*-tests for dependent samples (performed using Statistica version 7.0; Statsoft, Tulsa, OK, USA) were used to compare memory load effects (1- vs 2-back) on functional brain activations (pseudo *z-*values) associated with correct recognition effects (RC–New) at the between-group level. Statistical analyses performed on behavioural data were performed using Statistica version 7.0 (Statsoft).

## Results

### WM behavioural performance

We found a significant main effect of condition (1- and 2-back) for all three behavioural measures, Acc, RTs and RT CV (Acc: F(1,38)=136.08; RTs: F(1,38)=138.58 and CV: F(1,38)=17.16; all *P'*s<0.0002) but no effect of the group (all *P*>0.19) and no interaction between the factors (condition × group; all *P'*s>0.24; see Table 2 for details of the behavioural data). Thus, there were no behavioural differences between ASD and TD children, across dependent variables (Acc, RTs or CV). Subsequent least significant difference Fisher *post hoc* analyses demonstrated that both groups of children performed significantly better in the 1-back compared with the 2-back condition (all *P'*s>0.003).

Likewise, no effect of the group (*P*>0.19) or interaction were found on the standardized subscales scores of the WMTB-C (subscales of the WMTB-C × group; *P*>0.55). We found a main effect of subscales of the WMTB-C (F(4,148)=8.73, *P*<0.00001) suggesting that regardless of the group of children, WM performance differed between subtests, with better performance for Digit Recall and Listening Recall than for Backward Digit Recall, Block Recall and Mazes Memory (all *P'*s<0.016), which otherwise did not differ from each other (all *P'*s>0.23) as demonstrated by least significant difference Fisher's *post hoc* analyses.

### MEG results

#### Within-group working memory brain processes in TD and ASD Children

Significant within-group activations associated with the correct recognition effect, where event-related responses associated with RC trials were significantly greater than the encoding (new) baseline trials (all *P'*s<0.05, corrected), are listed in Table 3 for the 1-back and in Table 4 for the 2-back condition.

1-Back correct recognition effect in TD and ASD children: In TD children, correct recognition effects (RC>New; P^corr^<0.05) were strongly associated with increased activity in the right medial temporal gyrus (375–425 ms) and large and sustained activity in the right hippocampus from 400 to 500 ms. The right precentral gyrus was also activated from 375 to 450 ms as well as the right medial prefrontal cortex (mPFC) from 400 to 450 ms. From 450 to 500 ms, TD children recruited the right middle cingulate cortex (MCC) followed by the ACC and the right orbito-frontal region, both marked by persistent long-lasting activity from 450 to 525 ms and 575 ms, respectively.

Within-group comparison (RC>New; *P*^corr^<0.05) conducted on recognition effects in ASD children revealed activations first in the left dlPFC from 200 to 275 ms, followed by the right mPFC from 400 to 500 ms. Large, long-lasting activity occurred then from 425 to 500 ms bilaterally in the insulae while the right hippocampus was activated from 450 to 500 ms. Finally, children with ASD recruited the left orbito-frontal gyrus and the ACC from 500 to 550 ms, as well as the right MCC from 525 to 575 ms.

2-Back correct recognition effect in TD and ASD children: Within-group comparison (RC>New; *P*^corr^<0.05) conducted on recognition effects in TD children in the 2-back condition revealed increased activity from 225 to 275 ms in the left insula and the left mPFC from 250 to 300 ms. From 325 to 375 ms, TD children recruited first the left intra-parietal sulcus (IPS) and the left dlPFC from 375 to 425 ms, followed by sustained activity in the right MCC from 425 to 500 ms.

In children with ASD, correct recognition effects (RC>New; *P*^corr^<0.05) were first associated with activity of the left mPFC from 200 to 250 ms and large activity of the left angular gyrus from 250 to 325 ms. We then observed activity in the left dlPFC from 275 to 325 ms, followed by large activity in the left precuneus from 325 to 400 ms as well as sustained activity in the right MCC from 475 to 525 ms.

Between load (1- vs 2-back) correct recognition effects in TD and ASD children: *T*-tests for dependent samples performed on recognition effects (RC—New) in TD children showed that the right hippocampus (from 400 to 475 ms) and the left ACC (from 450 to 500 ms) elicited stronger activation during the 1- than the 2-back conditions (all *P*'s<0.01). Whereas they recruited the left insula (from 225 to 275 ms) and the IPS (from 325 to 375 ms) more during the 2-back than the 1-back condition (all *P'*s<0.03).

Similar comparisons performed in children with ASD revealed more activation in the left dlPFC (*P*=0.05) and the bilateral insulae (all *P'*s<0.01) in the 1- than the 2-back conditions from 225 to 500 ms, whereas in the high-memory load condition children with ASD showed more activity in the left precuneus (from 325 to 375 ms) compared with the low-memory load condition (*P*<0.03; see Supplementary Table S1 in Supplementary Information for details).

#### Comparison of WM brain processes between TD and ASD children

Between-group comparisons performed on recognition-related brain activations observed in the within-group analyses revealed overlapping but also different WM networks in ASD and TD children both in the higher- (2-back) and lower- (1-back) memory load conditions (see Tables 3 and 4, in bold).

**Figure 2:**
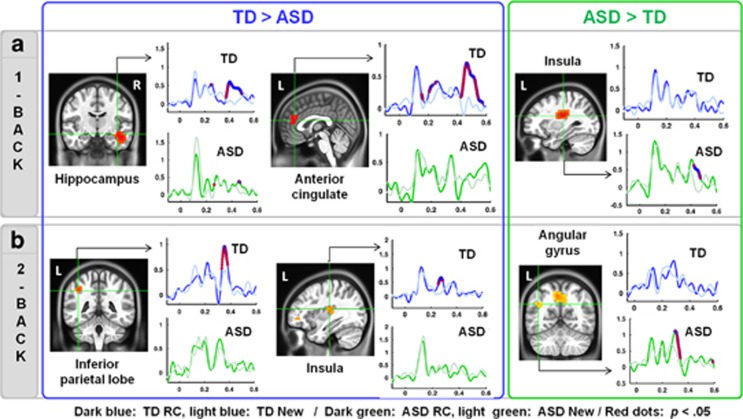
WM-related brain differences between TD and ASD children in (**a**) the 1-back and (**b**) the 2-back conditions. Brain images: significant brain activations associated with correct recognition effects (RC>New, *P*<0.5 corrected) that were stronger in TD children than children with ASD (left panel in blue) or stronger in children with ASD than in TD children (right panel in green, all *P*<0.005). Each brain image is associated with two overlaid time-course plots (*y* axes: pseudo *z-*values; *x* axes: time in seconds) representing statistical comparisons (*P*<0.5, red dots) between virtual sensors associated with RC (dark blue in TD and dark green in ASD children) and New (light blue in TD and light green in ASD children) trials. ASD, autism spectrum disorder; RC, repeat–correct' trial; TD, typically developing children.

1-Back-related functional brain differences between TD and ASD children: Between-group comparison (see Figure 2a) performed on brain areas associated with correct recognition effects in the 1-back condition revealed important differences in five brain regions: the right hippocampus, the left dlPFC, the bilateral insulae, the ACC and the MCC. Children with ASD showed significantly stronger activity in the left dlPFC from 200 to 275 ms and in the bilateral insulae from 425 to 500 ms than TD children. Conversely, from 400 to 500 ms, TD children showed long-lasting activation in the right hippocampus where children with ASD only recruited this region at the same level as controls from 450 to 500 ms. Moreover, both the ACC and MCC were significantly more active from 450 to 500 ms in TD children than children with ASD.

2-Back-related functional brain differences between TD and ASD children: Between-group comparison (Figure 2b) of brain areas associated with correct recognition effects in the 2-back condition also revealed significant differences in WM brain processes in five regions. Children with ASD showed less activation in the left insula (from 225 to 275 ms), the left IPS (from 325 to 375 ms) and the right MCC (from 425 to 500 ms) than TD children. By contrast, children with ASD showed stronger WM-related activations of the left angular gyrus (from 250 to 325 ms) and of the left precuneus (from 325 to 400 ms).

### Correlation analyses

**Figure 3:**
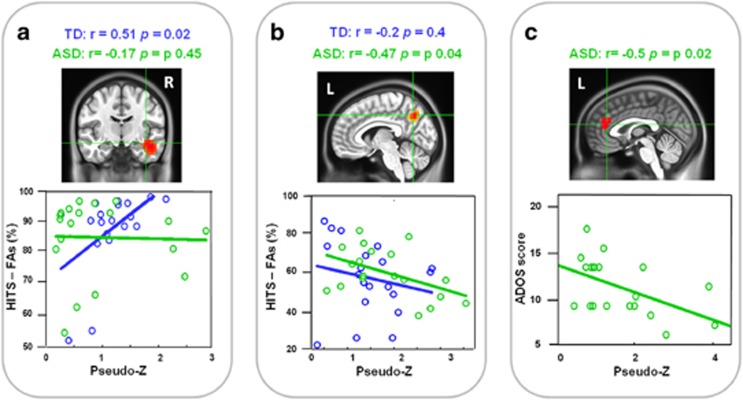
Significant correlation coefficients (all *P*<0.05) between event-related MEG activity in (**a**) the right hippocampus (1-back; from 450 to 500 ms) and (**b**) the left precuneus (2-back; from 325 to 375 ms), and behavioural performance in TD (blue) and ASD (green) children. (**c**) Significant correlation coefficient (*P*<0.05) between event-related MEG activity in ACC (1-back; from 450 to 500 ms) and ASD symptom severity in ASD. ACC, anterior cingulate cortex; ASD, autism spectrum disorder; FA, false alarm; MEG, magnetoencephalography; TD, typically developing children.

Data inspection revealed a significant positive correlation coefficient between the right hippocampal activity and performance (that is, percentage of HITS−percentage of FA) in TD but not in ASD children (average *r*=+0.51; *P*=0.02 in TD children vs *r*=−0.17; *P*=0.45 in ASD children, see Figure 3a) during the 1-back condition. 2-Back-related activity in the left precuneus was a positively correlated performance (that is, percentage of HITS−percentage of FA) in ASD but not in TD children (average *r*=−0.2; *P*=0.4 in TD children vs *r*=−0.47; *P*=0.04 in ASD children, see Figure 3b). Finally, we observed a significant negative correlation between the ACC activity during the 1-back task and the severity of autistic symptoms assessed through the ADOS scores (average *r*=−0. 5; *P*=0.02 in ASD children, see Figure 3c). This last result indicates that the more severe the symptoms were in the children with ASD, the less they activated the ACC during the 1-back task.

## Discussion

Our study, particularly in relation to the sequence of activations revealed using the temporal resolution of MEG, highlights atypical WM-related brain processes in children with ASD. These atypicalities showed significant, qualitative functional brain differences, where the groups recruited distinct brain regions to perform the WM task, as well as quantitative differences where both groups activated the same region but to a differing extent. Cerebral functional dissimilarities occurred despite any behavioural differences in performance between ASD and TD children, consistent with most results in the literature (for example, see Ozonoff and Strayer,^18^ Russell *et al.*^45^ and Griffith *et al.*^46^). However, six children with ASD but only one TD child included in the original sample of participants tested, performed the task at the chance level. Among them, five ASD were unable to do the 2-back condition strengthening the hypothesis that behavioural WM impairments tend to appear in ASD in higher-memory load and/or complex conditions.^2, 12, 47, 48^

### Fronto-insular vs hippocampal WM-related brain differences

In the 1-back condition, children with ASD showed reduced WM-related activity of the right hippocampus from 400 to 450 ms and of the ACC and the MCC from 450 to 500 ms. In contrast, WM processes during the 1-back condition were associated with stronger activity of left dlPFC from 200 to 275 ms and of the insulae bilaterally from 425 to 500 ms in children with ASD compared with controls. Interestingly, in TD children, both the right hippocampus and the MCC were more active during the low- than the high-memory load condition. These results demonstrate qualitative group differences in the WM brain processes underlying the lower WM cognitive load condition, as recognition effects did not rely on either the insula or the dlPFC recruited in TD children during the 1-back condition.

The central role of the right hippocampus in the 1-back WM task in TD children was further strengthened by the presence of a positive correlation between increased activity in this region and improved behavioural performance (percentage of HITS−percentage of FAs), whereas a similar correlation was not present in children with ASD. Moreover, although with different timing, the recruitment of the hippocampus during the 1-back condition is concordant with an MEG report in adults where 1-back recognition processes were associated with increased activity in the right hippocampus from 150 to 200 ms.^33^ In the TD children, correct recognition effects were associated with an increased activity in the hippocampus from 400 to 500 ms, demonstrating longer processing time in TD children than adults in this region. Reduced functional activity in the hippocampus observed in children with ASD might be related to reports of structural hippocampal abnormalities in this population^49, 50^ and suggest impaired hippocampus-related WM processing in ASD. Both neuroimaging and patient studies have highlighted the relation between the hippocampi and WM with associations between medial temporal-lobe damage and impaired WM.^51, 52, 53, 54, 55^ Accumulating evidence suggests that the right hippocampus is involved in the rapid binding and storage of cortical inputs into a coherent memory representation and, therefore, has a crucial role in transferring a short-term store into a durable long-term representation.^56, 57, 58, 59, 60^ Moreover, it has been shown that right hippocampal activity predicts successful long-term memory for stimulus pairs that were correctly classified in a WM task.^61^

Neither the left dlPFC nor the bilateral insulae that were significantly more active during the 1-back task in ASD than in TD children, have been specifically implicated in long-term memory binding processes in the literature. Thus, if this fronto-insular network helped ASD children to perform the *n*-back task in our study, we expect that this network may not support long-term memory consolidation processes adequately in other situations, eventually leading to memory and/or cognitive difficulties in ASD. The 1-back-related qualitative differences involving the insulae and the left dlPFC in children with ASD but not in TD children were impressive. Both regions have been associated with increased task difficulty and in helping in monitoring and the maintenance of newly encoded representations,^28, 62^ as well as cognitive control (see below), respectively, in the context of various WM tasks. Using an oculomotor WM task, Scherf *et al.*^63^ showed that weaker performance in children was correlated with higher activity in the insula compared with adults' performance, which relied instead on distributed brain areas including the temporal lobe. This fMRI study also showed that WM-related dlPFC activity increased from childhood (10–13 years) to adolescence (14–17 years) reflecting improved performance, but then decreased from adolescence to adulthood when performance was similar; this was interpreted as reflecting greater effort in adolescents. Insula, especially when right-lateralized, has been found consistently to be activated in cognitively demanding^64^ and/or challenging task conditions (see Sridharan *et al.*^65^). The insula has been described as a network hub that helps switch among brain systems, especially by engaging attentional, WM and higher-order control brain processes while disengaging other systems in challenging task conditions.^65, 66^ The fact that TD children activated the left insula (and left dlPFC, but to the same extent as ASD children) not in the 1-back but in the 2-back condition, strengthens the interpretation that these regions are recruited in WM conditions of higher complexity. However, compared with controls, children with ASD showed reduced activity of the left insula during the 2-back condition, suggesting that with higher loads on the WM memory task, children with ASD failed to recruit this region to support their performance. This interpretation was strengthened by the fact that, whereas TD children recruited the left insula more during the 2-back than the 1-back condition, the opposite effect was found in the ASD group who recruited the insulae more during the 1-back than the 2-back condition.

### Intra-parietal WM-related differences

In both groups of children, correct recognition effects partly relied on inferior parietal regions during the 2-back condition. However, the specific recruitment of the left angular gyrus (from 250 to 325 ms) and the left precuneus (from 325 to 400 ms) in children with ASD instead of the left insula (from 225 to 275 ms) and the left IPS (from 325 to 375 ms) in controls showed that WM-related brain processes qualitatively differed between groups, as seen in the low-memory load condition.

Reduced activity of WM-related left IPL and, in particular, lower-connectivity processes between the left IPS and the cingulate cortex have been reported in ASD using a WM fMRI study using a single-letter *n*-back paradigm;^21^ structural abnormalities in the left inferior parietal cortex have been related to attention deficits in children with ASD and attention deficit hyperactivity disorder.^67^ IPL activity is associated with the ability to complete WM tasks in healthy children^29, 30, 31, 32^ and is recognized as a key region for *n*-back WM tasks in a range of fMRI studies.^68^ IPL has an important role in attention and spatial processing and hence, has been related to more sophisticated levels of WM performance.^63^ Interestingly, the literature indicates a clear and reliable dissociation between the dorsal parietal cortex (including the IPS) being described as the dorsal ‘top-down/executive' (non-automatic, goal directed and having high-executive demands) and the ventral parietal cortex (including the angular and supramarginal gyri), associated with ventral ‘bottom-up/automatic' processes across multiple domains beyond attention and episodic memory (see Humphreys and Lambon Ralph^69^ for a recent meta-analysis). According to this view, the involvement of the dorsal parietal cortex (that is, IPS) in controls and the ventral angular gyrus in children with ASD reflects the recruitment of different brain processes and functional strategies to reach a similar level of performance.

We suggest that the recruitment of the left precuneus in children with ASD also contributes to WM performance. The precuneus has been related to children's limited access to inferior parietal regions^63^ and is often activated in cognitively demanding tasks requiring enhanced voluntary attention^70^ and increased memory load conditions.^71, 72^ This interpretation is reinforced by the observation of a negative correlation between performance (that is, percentage of HITS−percentage of FA) and increased activity in the left precuneus in children with ASD, but not in TD children. Moreover, complementary analyses showed that children with ASD tended to recruit this region more in the high- compared with the low-memory load condition. The precuneus is also a hub region that allows the monitoring of cognitive functions (for example, see Hagmann *et al.*^73^and Misic *et al.*^74^) and is involved in a variety of processing, including visuo-spatial imagery, visual attention and episodic memory retrieval.^75^ Future research is needed to understand the functional impact of using these different regions (insulae-IPS vs angular gyrus-precuneus) and their possible specific relations with WM processes in children with ASD.

### Frontal-related WM brain differences

Between-group differences observed in the frontal areas were mainly quantitative. In both groups, late-sustained event-related components were activated in the cingulate cortices but to a lesser extent in children with ASD. Delayed activations were observed both in the ACC and MCC in children with ASD (500–550 and 525–575 ms, respectively) compared with TD children (450–525 and 450–500 ms, respectively) in the 1-back condition. Moreover, activity in the MCC was also delayed in children with ASD (475–525 ms) compared with TD children (425–500 ms) during the 2-back condition. Reduced activity in the MCC has been related to functional deficits of the attentional networks in autism and correlated with increased impairments in communication and language abilities in children with ASD.^76^

Although contradictory with some data,^24, 25, 26^ atypical WM-related activity in the ACC in ASD is reminiscent of a previous fMRI study conducted in adolescents with ASD.^23^ Moreover, alterations in activity in the cingulate, demonstrated by reduced glucose metabolism^77^ or disrupted white matter,^78^ have been shown throughout the anterior, mid- and posterior cingulate cortex in patients with autism. The crucial impact of atypical ACC activity in the symptomatology of ASD is strengthened by our results. We found a significant correlation between reduced activity in the ACC and an increase in autistic symptoms severity in our population of children with ASD. Therefore, given the critical role of the anterior and midcingulate cortex in attentional circuits that help the regulation of both cognitive and emotional processing (for a review see Bush *et al.*^79^), we suggest that future research investigates the specific associations of cingulate-related WM functions and their relations with social and communication deficits in ASD.

### Potential impact on social deficits of autism

Our results suggest that, although different brain networks were able to support similar WM behavioural performance in TD and ASD children, in the context of the *n*-back task, the neurocognitive strategies observed in children with ASD might also affect the processing of complex social situations. Executive functions including WM have been implicated in the processing of social information^61, 80^ requiring, for instance, theory of mind skills^81^ that are reported impaired or delayed in ASD.^82, 83, 84^ Accordingly, our results identified abnormal brain activity in the ACC, the MCC, the insula, the medial temporal lobe and the inferior parietal lobe that have been involved in high-level executive and social cognition processes.^85^ Moreover, the atypical functioning of the ACC and the insula in the context of cognitively challenging situations has been considered as a key aspect of psychopathology in several neurological and psychiatric disorders, including fronto-temporal dementia, autism and anxiety disorders.^86, 87, 88^ Thus, our results suggest that the abnormal spatiotemporal brain processes identified in children with ASD, both in the low- and high-cognitive load WM conditions, may contribute to the social deficits of autism.

## Figures and Tables

**Table 1:** Demographic information

		*Autism (20)*	*Control (20)*		P*-values*
Age (years)	Mean±s.d.	11.25±1.58	11.26±1.64	*t* (38)=−0.02	0.97
IQ	Mean±s.d.	108.25±14.31	115.95±10.97	*t* (38)=−1.9	0.06
Sex	Male:female	16:4	13:7	*χ*^2^(1)=1.12	0.28
Handedness	Right:left	18:2	17:3	*χ*^2^(1)=0.22	0.63

Abbreviation: IQ, intelligence quotient.

**Table 2:** Mean behavioural performance on the *n*-back task

	*ASD (20)*	*TD (20)*	
*1-Back*			
* *Acc (%)	90.06±12.51	92.36±10.87	NS
* *RTs (s)	0.51±0.07	0.48±0.07	NS
* V* (%)	0.33±0.04	0.33±0.06	NS
			
*2-Back*			
* *Acc (%)	60.58±15.32	68.17±16.66	NS
* *RTs (s)	0.64±0.1	0.61±0.09	NS
* V* (%)	0.40±0.1	0.39±0.06	NS

Abbreviations: Acc, accuracy; ASD, autism spectrum disorder; NS, not significant; RT, reaction time; TD, typically developing; V, coefficient of variability.

Values are denoted as mean±s.d.

**Table 3:** Areas of activation associated with correct recognition effect (RC>New) in the 1-back memory load in TD and ASD children

*Within-group recognition effects*							*Group differences*
*Time windows*		*Brain area*	*Pseudo* z*-values*	*MNI coordinates*			
*TD children*							
375–425	R	MTG	0.75	44	−7	−18	
	R	Precentral gyrus	0.66	44	8	27	
**400–450**	**R**	**Hippocampus**	**0.87**	**42**	−**12**	−**18**	**TD>ASD**
	R	Precentral gyrus	0.57	49	8	27	
	R	mPFC	0.54	19	48	2	
**425–475**	**R**	**Hippocampus**	**0.74**	**39**	−**12**	−**18**	**TD>ASD**
**450–500**	R	Hippocampus	0.52	44	−12	−18	
	**R**	**MCC**	**0.52**	**4**	**−27**	**42**	**TD>ASD**
	R	OFG	0.62	9	28	−23	
	**L**	**ACC**	**0.73**	**−1**	**38**	**22**	**TD>ASD**
475–525	R	ACC	0.72	4	38	7	
525–575	R	OFG	0.47	24	23	−18	
							
*ASD children*							
**200–250**	L	**dlPFC**	**0.52**	−**26**	**18**	**37**	**ASD>TD**
**225–275**	L	**dlPFC**	**0.64**	−**21**	**23**	**37**	**ASD>TD**
400–450	R	mPFC	0.89	29	53	7	
**425–475**	L	**Insula**	0.84	−31	−22	22	**ASD>TD**
	R	**Insula**	0.83	34	18	7	**ASD>TD**
**450–500**	R	**Insula**	0.73	39	−2	12	**ASD>TD**
	R	Hippocampus	0.73	29	−17	−13	
	R	mPFC	0.78	29	53	7	
500–550	L	OFG	0.61	−31	43	−3	
	L	ACC	0.61	−1	43	22	
525–575	R	MCC	0.66	14	−27	47	

Abbreviations: ACC, anterior cingulate cortex; ASD, autism spectrum disorder; dlPFC, dorso-lateral prefrontal cortex; L, left; MCC, middle cingulated cortex; MNI, Montreal Neurological Institute; mPFC, medial prefrontal cortex; MTG, medial temporal gyrus; OFG, orbito-frontal gyrus; PCG, precentral gyrus; R, right; RC, repeat–correct' trial; TD, typically developing.

Note: Statistical significance was set for the significant voxels of activation at *P*<0.05, corrected for multiple comparisons in the whole source–space volume for within-group comparisons (recognition effect in each group; RC vs New stimuli) and at *P*<0.005 uncorrected for between-group comparisons (recognition contrast (RC–New stimuli) by group, highlighted in bold).

**Table 4:** Areas of activation associated with correct recognition effect (RC>New) in the 2-back memory load in TD and ASD children

*Within-group recognition effects*							*Group differences*
*Time windows*		*Brain area*	*Pseudo* z*-values*	*MNI coordinates*			
*TD children*							
** 225–275**	**L**	**Insula**	**0.47**	**−31**	**−22**	**12**	**TD>ASD**
** **250–300	L	mPFC	0.57	−26	43	2	
** 325–375**	**L**	**IPS**	**0.46**	**−41**	**−47**	**42**	**TD>ASD**
** **375–425	L	dlPFC	0.59	−41	18	27	
** 425–475**	**R**	**MCC**	**0.46**	**4**	**−12**	**47**	**TD>ASD**
** 450–500**	**R**	**MCC**	**0.43**	**4**	**−2**	**42**	**TD>ASD**
							
*ASD children*							
** **200–250	L	mPFC	0.49	−11	48	22	
** 250–300**	**L**	**Angular**	**0.46**	**−41**	**−62**	**32**	**ASD>TD**
** 275–325**	L	**Angular**	**0.52**	**−41**	**−52**	**32**	**ASD>TD**
	L	dlPFC	0.44	−46	43	7	
** 325–375**	L	**Precuneus**	**0.41**	**−6**	**−67**	**37**	**ASD>TD**
** 350–400**	L	**Precuneus**	**0.5**	**−6**	**−67**	**42**	**ASD>TD**
** **475–525	R	MCC	0.42	4	13	47	
	R	MCC	0.42	−1	−2	57	

Abbreviations: ASD, autism spectrum disorder; dlPFC, dorso-lateral prefrontal cortex; IPS, intra-parietal sulcus; MCC, middle cingulated cortex; MNI, Montreal Neurological Institute; mPFC, medial prefrontal cortex; RC, repeat–correct' trial; TD, typically developing.

Note: statistical significance was set for the significant voxels of activation at *P*<0.05, corrected for multiple comparisons in the whole source–space volume for within-group comparisons (recognition effect in each group; RC vs New stimuli) and at *P*<0.005 uncorrected for between-group comparisons (recognition contrast (RC–New stimuli) by group, highlighted in bold).
