# Supplementary material for: Atypical spatiotemporal signatures of working memory brain processes in autism
Source: Transl Psychiatry. 2015 Aug 11;5(8):e617–. doi: 10.1038/tp.2015.107 (PMC4564562; doi:10.1038/tp.2015.107)
Supplement: Supplementary Information [file tp2015107x1.doc]

| **Supplemental table S1** | | | | | | | | | |
| --- | --- | --- | --- | --- | --- | --- | --- | --- | --- |
|  |  |  |  |  |  |  |  |  |  |
|  | Timing |  | area | MNI Coordinates (x,y,z) | 1-back | 2-back | T(19) | p-values | Load effect |
|  |  |  |  |  | pseudo-z values:  (mean ±SD) | |  |  |  |
| TD >ASD |  |  |  |  |  | |  |  |  |
|  | 400-450 | R | HPC | 42 -12 -18 | .84 ±.78 | .28±.66 | 3.09 | **.005** | **1>2b** |
|  | 425-475 | R | HPC | 39 -12 -18 | .74  ±.61 | .22  ±.68 | 2.7 | **.01** | **1>2b** |
|  | 450 500 | R | MCC | 4 -24 42 | .52±.53 | .31±.53 | 1.06 | .29 | 1=2b |
|  |  | L | ACC | -1 38 22 | .73±.58 | .43±.44 | 1.94 | **.06** | **1≥2b** |
|  | 225-275 | L | Insula | -31 -22 12 | .15±.45 | .47±.37 | -2.3 | **.03** | **2>1** |
|  | 325-375 | L | IPS | -41 47 42 | .02±.41 | .46±.4 | -7.19 | **.000001** | **2>1** |
|  | 425-475 | R | MCC | 4 -12 47 | .56±.5 | .46±.63 | .50 | .62 | 2=1 |
|  | 450-500 | R | MCC | 4 -2 42 | .53±.54 | .42±.56 | .54 | .59 | 2=1 |
|  |  |  |  |  |  |  |  |  |  |
| ASD>TD |  |  |  |  |  |  |  |  |  |
|  | 200 250 | L | dlPFC | -26 18 37 | .52±.52 | .27±.54 | 1.52 | .14 | 1=2b |
|  | 225-275 | L | dlPFC | -21 23 37 | .64±.61 | .22±.55 | 2.08 | **.05** | **1≥2b** |
|  | 425-475 | L | Insula | -31 -22 22 | .84±.64 | .22±.4 | 4.2 | **.0004** | **1>2b** |
|  |  | R | Insula | 34 18 7 | .83±.85 | .13±.45 | 2.96 | **.008** | **1>2b** |
|  | 450-500 | R | Insula | 39 -2 12 | .73±.79 | .18±.46 | 2.7 | **.01** | **1>2b** |
|  | 250-300 | L | Angular | -41 -62 32 | .42±.64 | .45±.55 | -.22 | .82 | 2=1 |
|  | 275-325 | L | Angular | -41 -52 32 | .37±.77 | .48±.61 | -.58 | .56 | 2=1 |
|  | 325-375 | L | Precuneus | -6 -67 37 | .13±.54 | .41±.49 | -2.29 | **.03** | **2>1** |
|  | 350-400 | L | Precuneus | -6 -67 42 | .32±.52 | .50±.50 | -1.52 | **.14** | **2≥1** |
|  | | | | | | | | | |

**Table S1. Between load (1- vs. 2-back) correct recognition effects in TD and ASD children.** T-tests for dependent samples were performed between load (1-vs.2-back) on functional brain activations (pseudo-z values) associated with correct recognition effects (RC –New) that differed between group (see also Table 3 and 4 of the manuscript in bold). HPC = Hippocampus; MCC = Middle cingulate cortex; ACC = Anterior cingulate cortex; IPS = Intra-parietal sulcus; dlPFC = dorso-lateral prefrontal cortex

Supplemental Figure S1

**
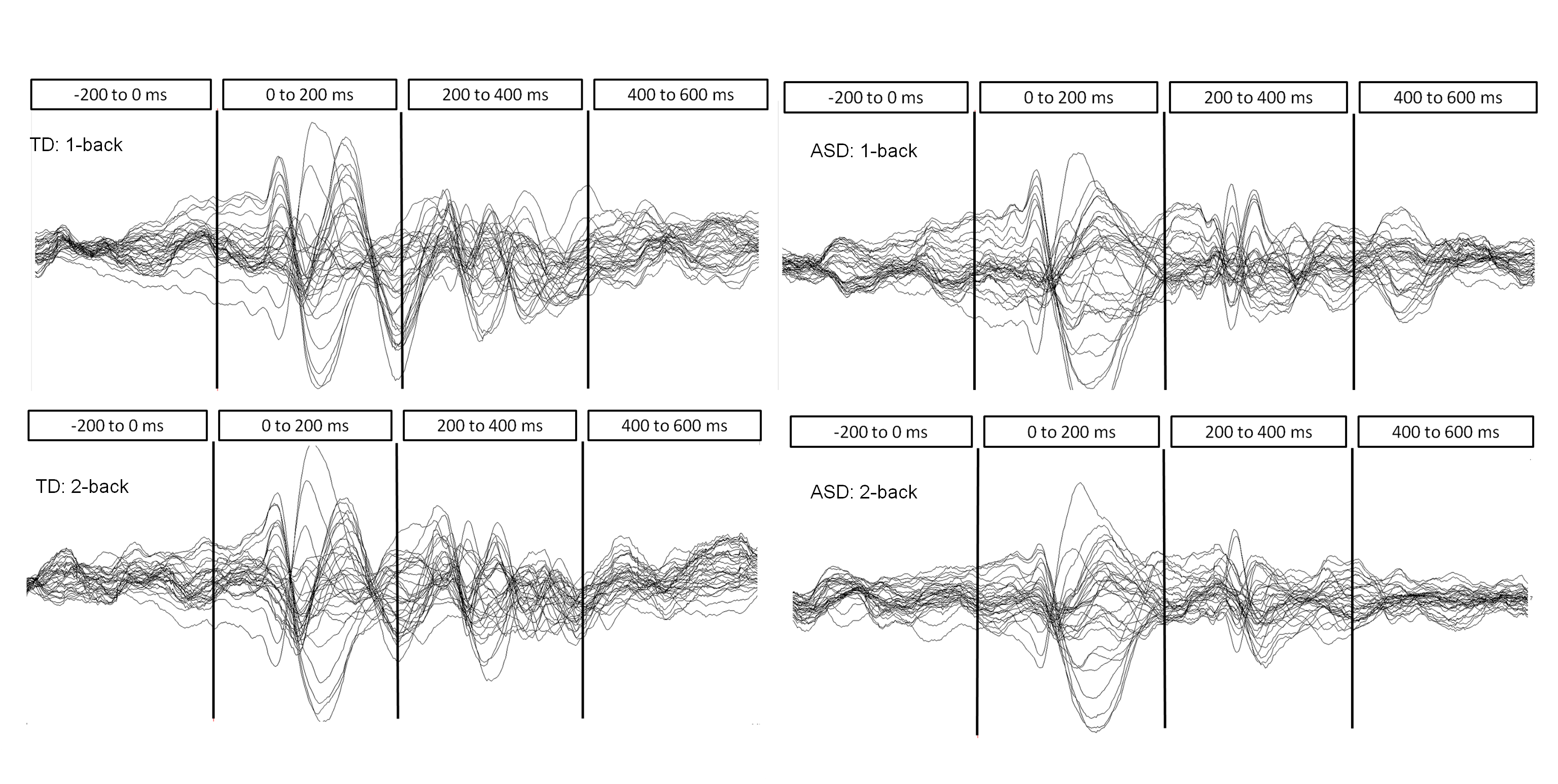
**

Figure S1. Grand average butterfly plots representing the Repeated correct (RC)-related evoked responses (from -200 to 1200 ms) at the sensor level in the 1-back and the 2-back conditions for the TD (left panel) and ASD (right panel) children.
